# New chemical mechanism explaining the breakdown of protective oxides on high temperature steels in biomass combustion and gasification plants†‡

**DOI:** 10.1039/c9ra00582j

**Published:** 2019-03-29

**Authors:** Tom Blomberg, Tripurari Tripathi, Maarit Karppinen

**Affiliations:** Aalto University, Department of Chemistry and Materials Science Kemistintie 1 02150 Espoo Finland tom.blomberg@aalto.fi tripurari.tripathi@aalto.fi maarit.karppinen@aalto.fi

## Abstract

Biomass is considered a replacement fuel over fossil fuels to mitigate climate change. The switch to biomass in the combustors changes the inorganic chemistry of the flue gases and leads to more severe corrosion of the construction materials of the combustors. The integrity of most high temperature steels relies on the formation of a protective Cr_2_O_3_ layer on the steel surface at a high temperature environment. The ash compound found on the heavily corroded steel in biomass combustion and gasification plants is KCl, but the mechanism, which triggers the breakdown of the protective Cr_2_O_3_ layer under the KCl salt is not known. We studied the chemical reactions involved with furnace exposure of KCl and KOH with Cr_2_O_3_ and identified the formed reaction products with XRD analysis. The amount of reaction products was analyzed from the leachates of the salt-oxide mixtures by UV/VIS spectroscopy. We also used thermodynamic Gibbs energy minimization calculations to evaluate the evolution of reactions as a function of temperature. The results suggests that the reaction of KCl with Cr_2_O_3_ involves a KOH reaction intermediate that forms before K_2_CrO_4_ is formed. The amount of reacted potassium as a function of temperature follows the trend of KCl decomposition to KOH and HCl(g) as predicted by thermodynamics calculations. Therefore, we argue that the suggested overall reaction of KCl with Cr_2_O_3_ as found in the corrosion literature: 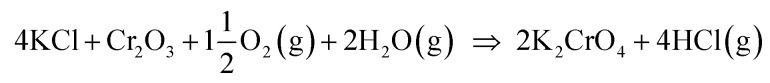, starting with the initiation step: KCl + H_2_O(g) ⇒ KOH + HCl(g) and then the formed KOH reacts with Cr_2_O_3_ to form K_2_CrO_4_. This explains the initial breakdown of the protective Cr_2_O_3_ under KCl salt in water containing high temperature atmospheres. The result is essential for the development of new alloys for biomass fired combustors.

## Abstract 2

New chemical mechanism explaining the breakdown of protective Cr_2_O_3_ on high temperature steels under KCl containing deposits in biomass combustion.

## Graphical abstract

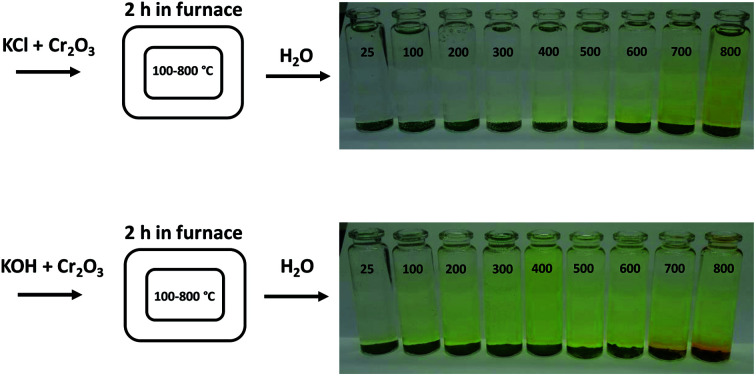

## 1. Introduction

Burning coal is responsible for approximately 25% of anthropogenic CO_2_ emissions. The largest use of coal as a fuel is in electricity and heat production, which accounts for about 16% of the total CO_2_ emissions.^1^ Replacing coal with sustainable biomass as the fuel in power boilers would lead to substantial reductions in the net CO_2_ emission. Also, SO_2_ emissions would be diminished due to the generally much lower sulphur content of biomass based fuels compared to coal. However, biomass combustion leads to more severe fouling and corrosion issues of the heat transfer surfaces in boilers.^2–5^ One way to minimize these problems is to operate the boilers with lower steam temperatures, but that lowers the efficiency of the Rankine cycle leading to higher emission factors per unit of electricity produced.

Potassium behaviour in the boiler has been identified as the major cause of slagging, fouling and corrosion in biomass fired boilers.^6^ Potassium is found associated in the fuel moisture as water soluble salts, or reacted with the organic functional groups (carboxylic, alkoxy, phenolic) of the lignocellulosic matrix.^7^ During combustion, potassium is released in the gas phase as elemental potassium, potassium chloride or potassium hydroxide as measured by mass spectrometric or optical methods.^8–10^ Due to its extreme reactivity, elemental potassium is expected to react to compounds in the vicinity of the biomass particle it is released from. Therefore, the major gas phase potassium compounds in the biomass boiler flue gases are KCl(g) and KOH(g). These compounds may react further with other fuel elements, such as silicon or sulphur to form potassium silicates or potassium sulphate. K_2_O–SiO_2_ silicate formation plays a major role in deposit formation in the furnace (slagging) and in bed agglomeration issues in fluidized bed boilers, where silica containing sand is often used as the bed material.^11–13^ Formation of K_2_SO_4_ plays a major role in aerosol and deposit formation further downstream of the boiler.^14,15^ K_2_SO_4_ formation may decrease the corrosion rate of the heat exchangers forming a less corrosive deposit than KCl or KOH/K_2_CO_3_. Its formation may however, also increase fouling rate of the heat exchangers forming tenuous deposits that are difficult to be removed by soot blowing, even though the corrosion rate under the deposits may still be low.^16,17^ During their path through the boiler, KCl(g) and KOH(g) in the flue gases condense out when the gas temperature is decreased below their dew points. Condensation can happen directly on the heat exchanger surfaces or on the ash particles present in the flue gas flow. Purely homogeneous nucleation is also possible in conditions where the flue gas does not contain enough foreign surfaces that can act as nucleation sites for heterogeneous nucleation.^18,19^ After condensation KCl(s,l) can react heterogeneously further to K_2_SO_4_(s,l) and KOH(s,l) can react to K_2_SO_4_(s,l), KCl(s,l) or K_2_CO_3_(s,l) as predicted by thermodynamic stabilities of the compounds.^20,21^

KCl induced corrosion of Fe–Cr steels has been studied extensively in the scientific literature.^22–28^ Recently, the effect of K_2_CO_3_ on the high temperature corrosion has gained more interest. It appears both potassium and chlorine are important in the corrosion reactions with steels. It has been suggested that potassium can initiate the destruction of the protective oxide, but chlorine is needed to sustain the corrosion.^29,30^ KOH induced corrosion has not been extensively studied in the context of high temperature corrosion in biomass fired boilers, but earlier studies have shown that Fe–Cr alloys are unsuitable for service in KOH containing high temperature environments.^31–33^ Earlier work of the author on the elemental balances of the deposit forming elements in biomass based fuels suggest that KOH(g) condensation may be more important in fouling and corrosion than has previously been thought. The details of K_2_CO_3_(s,l) formation on the heat exchanger surfaces has not been clarified yet, but its formation has been predicted by thermodynamics when the (Cl(g) + 2S(g)) molar content in the flue gases is lower than the molar K(g) content. K_2_CO_3_(s,l) is formed on the heat exchanger surfaces likely *via* a surface reaction of adsorbed KOH(ads.) with CO_2_(g). Homogeneous formation of K_2_CO_3_(g) in the gas phase, followed by condensation of K_2_CO_3_(g) is less likely, because of the thermodynamic instability of K_2_CO_3_(g).^34,35^ K_2_CO_3_(s,l) has also been directly detected in some boiler deposits.^17^ In this work we studied in detail the reactivity of KCl and KOH towards Cr_2_O_3_ and Fe_2_O_3_, the protective oxide components formed on the Fe–Cr alloys in high temperature oxidizing service conditions. The results may also be of interest for chromite ore roasting by KOH and for understanding corrosion of Cr_2_O_3_ containing refractory bricks in potassium and chlorine containing environments.^36–38^

## 2. Experimental

### 2.1 Preparation of the mixtures and furnace exposures

KOH–Cr_2_O_3_, KCl–Cr_2_O_3_, KOH–Fe_2_O_3_, KCl–Fe_2_O_3_ and K_2_CrO_4_–Cr_2_O_3_ mixtures were prepared by mixing known amounts of powders in a 10 ml glass bottle and manually shaking the bottles for approximately 30 s. KOH was from Sigma Aldrich technical grade ≥ 85%, KCl from Alfa Aesar, ACS grade 99–100.5%, K_2_CrO_4_ from Merck, EMSURE®, ACS grade ≥ 99.5%, Cr_2_O_3_ from E. Merck, grade unknown and Fe_2_O_3_ from Johnson Matthey Chemicals, Specpure® grade. All powders were weighted with Sartorius CPA225D analytical balance. Larger agglomerates of KCl, KOH and K_2_CrO_4_ were grinded manually in a mortar before mixing with the Cr_2_O_3_ or Fe_2_O_3_ powder. Cr_2_O_3_ and Fe_2_O_3_ powders seemed visually homogeneous without agglomerates and were used without any pre-grinding. KCl/Cr_2_O_3_, KCl/Fe_2_O_3_ or KOH/Cr_2_O_3_, KOH/Fe_2_O_3_ molar ratios were 1 and K_2_CrO_4_/Cr_2_O_3_ molar ratio was 0.5 in order to have the same K/metal molar ratio in all of the mixtures. A 10 g batch of each mixture was prepared in one go in a screw cap sealed bottle. From the 10.0 g batch bottles, 1.00 g samples were weighted to a 10 ml sintered Al_2_O_3_ crucible and then loaded immediately in a muffle furnace (Nabertherm P330) that was at the isothermal exposure temperature (100–800 °C). The samples were exposed in the furnace for 2 hours in ambient air atmosphere. After furnace exposures, the samples were cooled in ambient air so that handling of the crucible was possible with nitrile gloves (5–10 min) and then transferred to glass bottles that were sealed with screw caps. Then the samples were placed in a desiccator cabin for storage. During the analyses, the sample exposure times to ambient air before starting the analyses were minimized by opening the cap and preparing the sample from the bottle only just before starting the analysis. However, the XRD analysis took approximately an hour per sample, therefore possible reactions during the analysis with ambient air could not be completely eliminated. KOH especially is known to be highly hygroscopic and reactive towards CO_2_ during exposure to ambient air.

### 2.2 Qualitative determination of CrO_4_^2−^ and Cr_2_O_7_^2−^

20 ml glass bottles were filled almost full with ion exchanged water. Then 0.10 g of each sample was added to the already water filled bottle. The bottles were not agitated by any means in order to let the sample powder sediment to the bottom of the bottle by gravity. The samples were left to stand overnight. The characteristic yellow colour of the CrO_4_^2−^ ion started to appear immediately after adding the sample powder and the intensity of the colour increased with time. The sample bottles were then photographed to record the characteristic yellow colour of CrO_4_^2−^ and the orange colour of Cr_2_O_7_^2−^.

### 2.3 Qualitative determination of colourful Fe-complex ions

20 ml glass bottles were filled almost full with ion exchanged water. Then 0.10 g of each sample was added to the already water filled bottle. The bottles were not agitated by any means in order to let the sample powder sediment to the bottom of the bottle by gravity. The samples were let to stand still overnight. All the solutions were colourless indicating no signs of water soluble FeCl_3_ (yellow to orange) or K_2_FeO_4_ (FeO_4_^2−^ ion is purple) in the samples in concentrations high enough to be visible to the naked eye.

### 2.4 XRD measurements

XRD analysis was done with PANalytical X'pert Pro PW 3040/60 powder diffraction spectrometer with monochromated Cu K-alpha X-ray source (*α* = 1.5406 Å). Samples were first grinded manually in a mortar to make a visually homogeneous powder. Then the sample holder was filled evenly with the powder, pressed against a flat surface to level the sample surface with the sample holder top surface and then XRD *θ*–2*θ* scans were recorded from 10–90°. The automatic phase identification algorithm of the X'Pert HighScore Plus program was used for preliminary identification of the phases. Then the results were checked manually and the most likely crystalline phases where manually identified using the JCPDS cards. The XRD cards used for phase identifications were 00-021-0645 for KOH, 00-011-0655 for K_2_CO_3_·1.5H_2_O, 00-015-0365 for K_2_CrO_4_, 04-007-3113 for KCl, 01-078-5435 for Cr_2_O_3_, 04-012-4476 for K_2_Cr_2_O_7_, 01-085-0599 for Fe_2_O_3_, 00-039-0892 for KFeO_2_, 00-039-1106 for K_2_Fe_4_O_7_ and 01-078-6089 for K_1.75_Fe_1.25_O_4_.

### 2.5 UV/VIS spectroscopic measurements of CrO_4_^2−^

Approximately 0.20 g of sample powder was mixed with 20 ml of room temperature ion exchanged water. The solution was first stirred in a beaker with a magnetic stirrer for 15 min. Then the solution was filtered using qualitative filter paper, 410 (Cat. No. 516-0802 from VWR), the residue washed two times with 10 ml of room temperature ion exchanged water. Then the filtrates were transferred to 50 ml volumetric flasks and the flask was filled to the mark with ion exchanged water and mixed. The CrO_4_^2−^ concentrations of the filtrates were determined by UV/VIS spectroscopy using Shimadzu UV-2600 spectrophotometer and polymethylmethacrylate cuvettes. Absorbance at 372 nm was used for CrO_4_^2−^. It is not possible to determine Cr_2_O_7_^2−^ and CrO_4_^2−^ separately, because they are in equilibrium with each other in a water solution. This equilibrium depends on the pH and p[Cr] of the solution. With pH values > 6.7, the CrO_4_^2−^ ion is reported to be the stable form regardless of the Cr-concentration.^39^ Therefore, the K_2_Cr_2_O_7_ possibly present originally in the samples was detected as CrO_4_^2−^ and the concentration determined reflects the sum of K_2_CrO_4_ + K_2_Cr_2_O_7_ originally in the sample. The pH values of the filtrates were determined to be ≥7 with a pH indicator sticks (Fisher Scientific number 10642751). All the KCl based samples had pH = 7 and KOH based samples had pH = 7 in samples exposed at ≥ 500 °C and pH 8–12 in samples exposed at ≤400 °C. The higher pH with low temperature samples in case of KOH mixtures was caused by the KOH that was not reacted to K_2_CrO_4_, but formed K_2_CO_3_·1.5H_2_O during the furnace/ambient exposures. When dissolving in water, K_2_CO_3_·1.5H_2_O results in a basic solution. Concentration standards were prepared by dissolving known amounts of K_2_CrO_4_ powder to ion exchanged water in 50 ml volumetric flasks (ESI†). Then the absorbance of the standards were measured and linear concentration–absorbance curves were established. Then the absorbance of the sample filtrates were measured and the concentrations were determined using the standard curves. In cases where the sample had CrO_4_^2−^ concentration so high that the absorbance was higher than the standards, the sample was diluted with pure water in volumetric flasks with 1 : 10 or 1 : 20 dilution ratios, which ever was suitable to bring the absorbance value in between the standards. Molar ratios reacted to K_2_CrO_4_ and K_2_Cr_2_O_7_ were calculated as follows:1
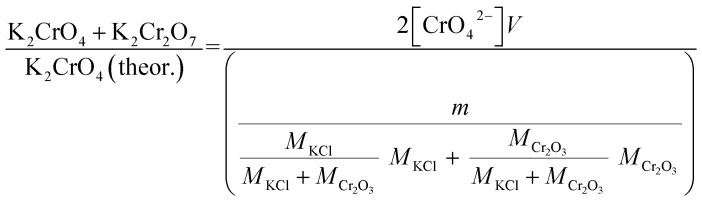
2
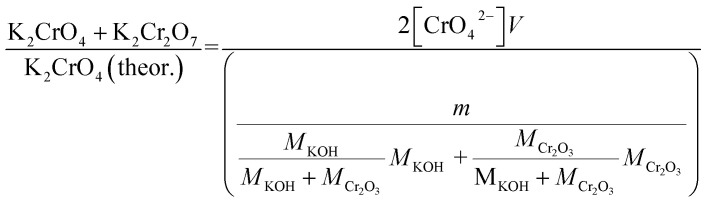
*V* = volume, l; *m* = sample mass, g; *M* = molar mass, g mol^−1^; [CrO_4_^2−^] = CrO_4_^2−^ ion concentration, mol l^−1^.

Error estimation of the above described method for K_2_CrO_4_ determination was done by running a few duplicate runs with KOH + Cr_2_O_3_ mixtures (2 at 300 and 400 °C exposure temperatures). Because of the small number of duplicate samples, error was estimated with the range rather than with statistical methods. The error range (max–min) of the method was found to be about 0.08 (or 8%-points). This error value was assumed to be similar at other exposure temperatures and also for the leaching tests described below for Fe^3+^.

### 2.6 UV/VIS spectroscopic measurements of Fe^3+^

#### 2.6.1 Water soluble Fe^3+^

Approximately 0.20 g of sample powder was mixed with 20 ml of room temperature ion exchanged water. The solution was stirred in a beaker with a magnetic stirrer for 15 min. Then the solution was filtered using qualitative filter paper 410 (Cat. No. 516-0802 from VWR), the residue washed two times with 10 ml of room temperature ion exchanged water. Then the filtrates were transferred to 50 ml volumetric flasks and the flask was filled to the mark with ion exchanged water and mixed. The Fe^3+^ concentrations of the filtrates were determined by UV/VIS spectroscopy using Shimadzu UV-2600 spectrophotometer. Absorbance at 225 nm was used for Fe^3+^. Fe^3+^ ion in a water solution is present as Fe^3+^, Fe(OH)^2+^ and Fe(OH)_2_^+^ ions or as non-water soluble Fe(OH)_3_ precipitate, depending on the pH of the solution.^40^ The pH values of the filtrates were determined with a pH indicator sticks (Fisher Scientific number 10642751). All the KCl based samples had pH = 7 and all the KOH based samples had pH = 12. The high pH value over the entire exposure temperature in case of KOH based samples indicate that they may have contained some unreacted KOH/K_2_CO_3_ or then the higher pH at firing temperatures ≥ 500 °C (which did not reveal any KOH/K_2_CO_3_ residues by XRD) was originated from the dissolution of KFeO_2_ (KFeO_2_ + 2H_2_O = K^+^ + Fe^3+^ + 4OH^−^). In order to shift the equilibrium so that all the dissolved Fe(iii) was in the Fe^3+^ state, the filtrates were buffered to pH = 1 by mixing 1 : 1 ratio of sample with a solution to 1 mol l^−1^ HCl (1 : 2 dilution). This pH stabilized solution was then used to fill the quartz cuvette in the UV/VIS absorption measurements. The added Cl-ion can also form complex ions with Fe in the form Fe(H_2_O)_6−*x*_(Cl)_*x*_^3−*x*^. Concentration standards were prepared by dissolving known amounts of FeCl_3_·6H_2_O powder to ion exchanged water and diluting (ESI†). The pH of the standards were buffered to 1 with 1 mol l^−1^ HCl before measurement as with the samples. Then the absorbance of the standards were measured and linear concentration–absorbance curves were established. Then the absorbance of the sample filtrates were measured and the concentrations were determined using the standard curves. Molar ratios reacted to KFeO_2_ were calculated as follows:3
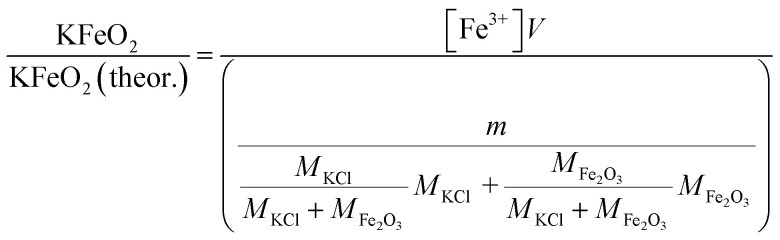
4
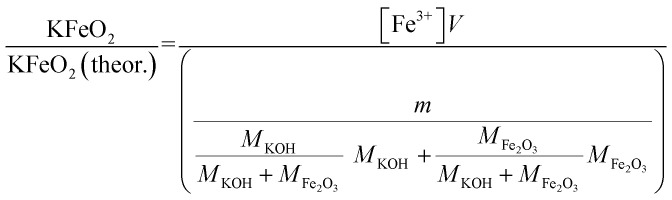
*V* = volume, l; *m* = sample mass, g; *M* = molar mass, g mol^−1^; [Fe^3+^] = Fe^3+^ ion concentration, mol l^−1^.

#### 2.6.2 Acid soluble Fe^3+^

The analysis was done in a similar way as has been discussed for the H_2_O soluble Fe^3+^ determination, except that the 15 min leaching steps were done in 20 ml of 1 mol l^−1^ HCl in water solutions instead of pure water. Also the washing of the filtration residue was done with HCl containing water (≈0.1 mol l^−1^ HCl, pH = 1) instead of pure water. This prevented the precipitation of Fe(OH)_3_ that was possible in the water leaching steps, where the pH values of the filtrates were ≥7. In case of 1 M HCl leaching, some filtrates had a yellow colour visible already to a naked eye, indicating that indeed the acid leaching resulted in much higher dissolution of the reaction products compared to H_2_O leaching. All the filtrates had a pH value of 1, measured with pH indicator sticks (Fisher Scientific number 10642751). Because the pH of the filtrates were stabilized to one already during the leaching step, they were used directly to fill the quartz cuvette in the UV/VIS absorption measurements. In cases where the sample had Fe^3+^ concentration so high that the absorbance was higher than the standards, the sample was diluted in volumetric flasks with 1 : 2, 1 : 50 or 1 : 100 dilution ratios, which ever was suitable to bring the absorbance value in between the standards. In the 1 : 50 and 1 : 100 dilution cases, the dilution was done by adding 1 ml of sample, then 5 ml of 1 mol l^−1^ HCl, and then filling the volumetric flask with pure H_2_O.

### 2.7 Thermodynamic calculations

HSC v6.12 Gibbs energy minimization software was used in the thermodynamic calculations.^41^ The input files for the calculations are presented in Table 1. Air composition^42^ with ≈0.99 mol% H_2_O vapor (RH ≈ 32% at 25 °C) was used to simulate the humid ambient gas phase in the muffle furnace. In case of KCl based systems, HCl(g) was added as the possible gas phase Cl-compound released in the reactions. Note that each gas phase component in the atmosphere has at least several times higher absolute amounts than any of the solid phase components. This assures that the amount of the formed products was never limited by the amount of the gas phase component, thus simulating an open ambient system. The calculated systems were kept as simple as possible. Therefore, in addition to the reactants, only the phases identified in the XRD analysis were added to the solid phase input file. In the case of Fe_2_O_3_ systems, K_2_Fe_4_O_7_ was detected by XRD in the sample with the highest exposure temperature with KOH, but unfortunately it was not found in the HSC v6.12 database and could not be included in the thermodynamic calculation.

**Table 1:** Input files in the thermodynamic equilibrium calculations with the HSC v.6.12 software^a^

KCl–Cr_2_O_3_	*T* [°C]	Amount [kmol]	Amount [mol%]	KCl–Fe_2_O_3_	*T* [°C]	Amount [kmol]	Amount [mol%]	KOH–Cr_2_O_3_	*T* [°C]	Amount [kmol]	Amount [mol%]	KOH–Fe_2_O_3_	*T* [°C]	Amount [kmol]	Amount [mol%]	KOH–KCl	*T* [°C]	Amount [kmol]	Amount [mol%]
Phase 1:		0.020	100.000	Phase 1:		0.020	100.000	Phase 1:		0.020	100.000	Phase 1:		0.020	100.000	Phase 1:		0.010	100.000
KCl	25.000	0.010	50.000	KCl	25.000	0.010	50.000	Cr_2_O_3_	25.000	0.010	50.000	Fe_2_O_3_	25.000	0.010	50.000	KCl	25.000	0.010	100.000
Cr_2_O_3_	25.000	0.010	50.000	Fe_2_O_3_	25.000	0.010	50.000	K_2_O*Cr_2_O_6_	25.000			KOH	25.000	0.010	50.000	KOH	25.000		
K_2_O*Cr_2_O_6_	25.000			KOH	25.000			KOH	25.000	0.010	50.000	K_2_CO_3_·1.5H_2_O	25.000			K_2_CO_3_	25.000		
KOH	25.000			K_2_CO_3_·1.5H_2_O	25.000			K_2_CO_3_·1.5H_2_O	25.000			KFeO_2_	25.000						
K_2_CO_3_·1.5H_2_O	25.000			KFeO_2_	25.000			K_2_CrO_4_	25.000										
K_2_CrO_4_	25.000																		
Phase 2:		99.996	100.000	Phase 2:		99.996	100.000	Phase 2:		99.996	100.000	Phase 2:		99.996	100.000	Phase 2:		99.995	100.000
H_2_O(g)	25.000	0.990	0.990	H_2_O(g)	25.000	0.990	0.990	H_2_O(g)	25.000	0.990	0.990	H_2_O(g)	25.000	0.990	0.990	H_2_O(g)	25.000	0.990	0.990
CO_2_(g)	25.000	0.035	0.035	CO_2_(g)	25.000	0.035	0.035	CO_2_(g)	25.000	0.035	0.035	CO_2_(g)	25.000	0.035	0.035	HCl(g)	25.000		
O_2_(g)	25.000	20.740	20.741	O_2_(g)	25.000	20.740	20.741	O_2_(g)	25.000	20.740	20.741	O_2_(g)	25.000	20.740	20.741	CO_2_(g)	25.000	0.035	0.035
N_2_(g)	25.000	77.311	77.314	N_2_(g)	25.000	77.311	77.314	N_2_(g)	25.000	77.311	77.314	N_2_(g)	25.000	77.311	77.314	O_2_(g)	25.000	20.740	20.741
HCl(g)	25.000			HCl(g)	25.000			Ar(g)	25.000	0.920	0.920	Ar(g)	25.000	0.920	0.920	N_2_(g)	25.000	77.310	77.314
Ar(g)	25.000	0.920	0.920	Ar(g)	25.000	0.920	0.920	KOH(g)	25.000			KOH(g)	25.000			Ar(g)	25.000	0.920	0.920
KCl(g)	25.000			KCl(g)	25.000											KOH(g)	25.000		
KOH(g)	25.000			KOH(g)	25.000											KCl(g)	25.000		

a K_2_Cr_2_O_7_ is marked as K_2_O*Cr_2_O_6_ in the software.

## 3. Results

### 3.1 KOH–Cr_2_O_3_ system

**Fig. 1:**
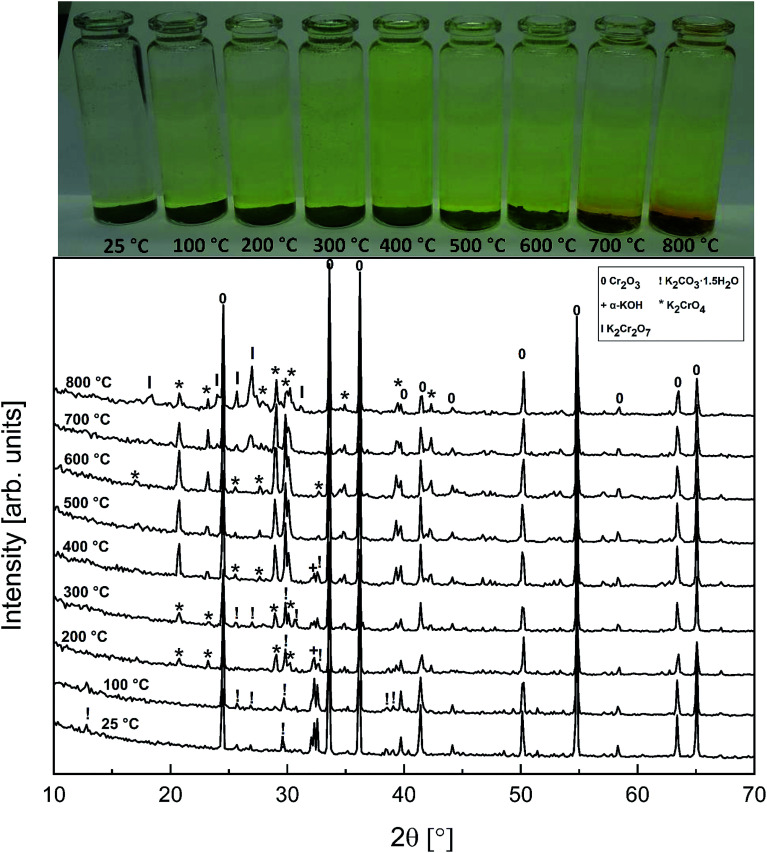
XRD patterns and appearance of the water solutions on top of the KOH–Cr_2_O_3_ mixtures after furnace exposures.

**Fig. 2:**
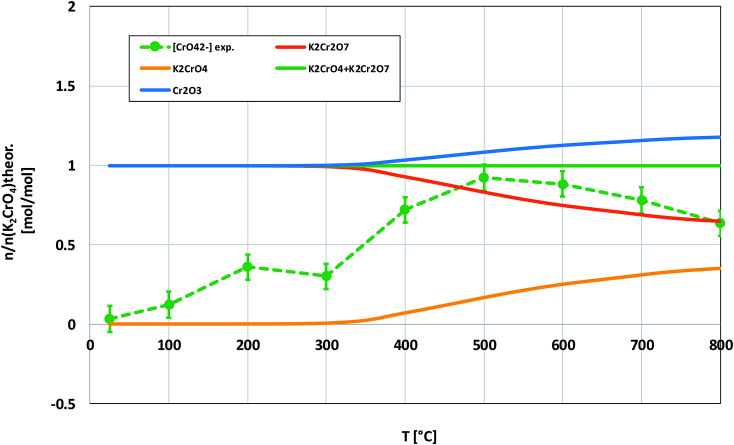
Comparison of the experimentally determined amount of [CrO_4_^2−^] in the filtrates with the thermodynamically predicted amount of the reaction products in the KOH–Cr_2_O_3_ system with pure water leaching.

The powder XRD analysis of the system after furnace exposures is shown in Fig. 1. It appears that KOH reacted partially with the air in the furnace and formed K_2_CO_3_·1.5 H_2_O in samples exposed to furnace temperatures below 500 °C. Samples exposed to 500 °C or higher firing temperatures did not contain any residual K_2_CO_3_·1.5 H_2_O or KOH. This was also reflected by the pH of the water soluble filtrates as explained in the experimental section. It seems that the reaction of KOH with Cr_2_O_3_, forming K_2_CrO_4_ is not fast enough below 500 °C and competes with K_2_CO_3_·1.5 H_2_O formation in the experimental conditions used. According to XRD analysis, K_2_CrO_4_ formation starts already with a solid–solid reaction at 200 °C. Melting point of KOH is 406 °C, so it appears that there is no need for molten phase to form in the system before K_2_CrO_4_ formation proceeds. The photograph in Fig. 1 reveals the characteristic colour of the CrO_4_^2−^ ion already appearing in the water soluble fraction of the products at room temperature exposures. However, this CrO_4_^2−^ may form from the dissolution of Cr_2_O_3_ in the basic water solution and therefore it is considered that the XRD analysis provides a better estimation of the onset temperature where K_2_CrO_4_ formation starts. The characteristic orange colour of the Cr_2_O_7_^2−^ ion was detected in samples fired at 700 °C and 800 °C. K_2_Cr_2_O_7_ was also detected in the XRD analysis at these firing temperatures. Thermodynamic calculations predict K_2_Cr_2_O_7_ to be the most stable reaction product throughout the temperature range used as shown in Fig. 2, but its formation appears to be kinetically prevented below 700 °C. The experimental results clearly show that K_2_CrO_4_ formation is kinetically preferred at the studied temperature range and exposure time used. Like explained above, K_2_CrO_4_ formation itself seemed also to be kinetically controlled at temperatures lower than 500 °C. The maximum CrO_4_^2−^ amount (>90% of the theoretical) in the reaction products was measured at 500 °C furnace exposure, after which the amount decreased slightly at higher temperatures. Taking into account the experimental errors associated with the CrO_4_^2−^ analysis, it is suggested that starting at 500 °C, practically all the KOH had reacted to K_2_CrO_4_ and that the slightly lower amounts of CrO_4_^2−^ detected at higher exposure temperatures can be explained by slight loss of KOH by evaporation, competing with the reaction with Cr_2_O_3_.

### 3.2 K_2_CrO_4_–Cr_2_O_3_ system

**Fig. 3:**
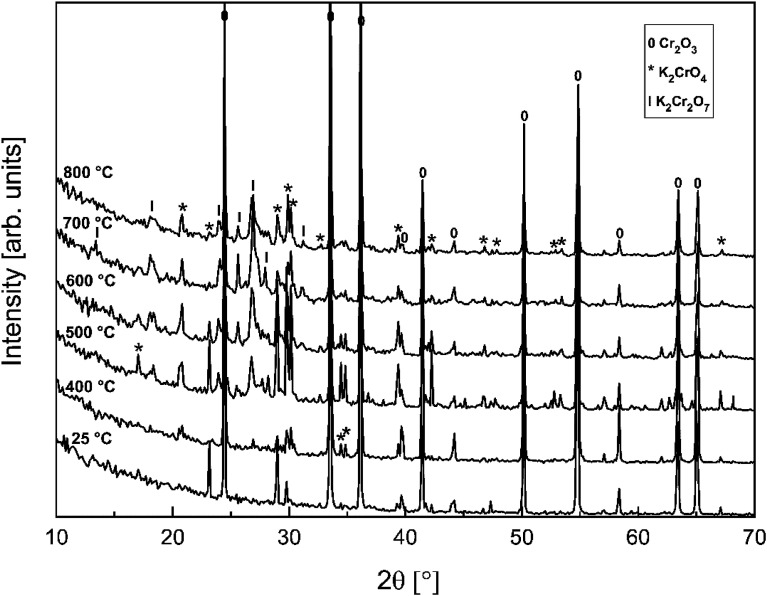
XRD patterns for the K_2_CrO_4_–Cr_2_O_3_ mixtures after furnace exposures.

In order to determine if the K_2_Cr_2_O_7_ formation at higher firing temperatures proceeds directly from the reaction with KOH or *via* first formation of K_2_CrO_4_ and then further reaction with Cr_2_O_3_, the K_2_CrO_4_–Cr_2_O_3_ system was studied with XRD analysis. Results are presented in Fig. 3. From the XRD analysis it is clear that K_2_CrO_4_ can react with Cr_2_O_3_ and form K_2_Cr_2_O_7_ at exposure temperatures ≥ 500 °C. Therefore, it is suggested that direct reaction of KOH with Cr_2_O_3_ forms K_2_CrO_4_ and that the formation of K_2_Cr_2_O_7_ requires always the K_2_CrO_4_ reaction intermediate to form first.

### 3.3 KCl–Cr_2_O_3_ system

**Fig. 4:**
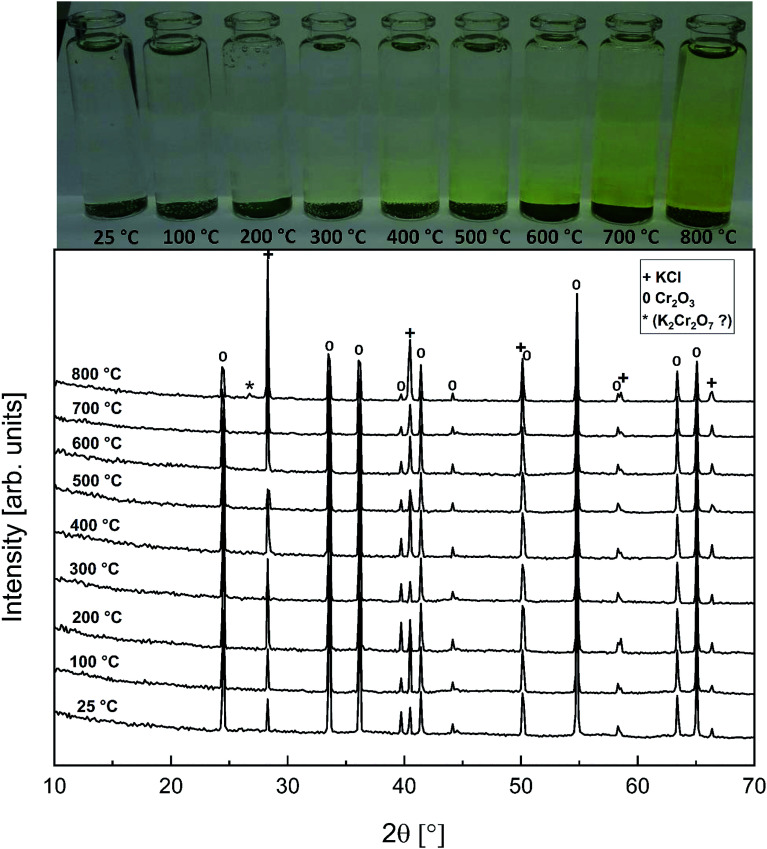
XRD patterns and appearance of the water solutions on top of the KCl–Cr_2_O_3_ mixtures after furnace exposures.

**Fig. 5:**
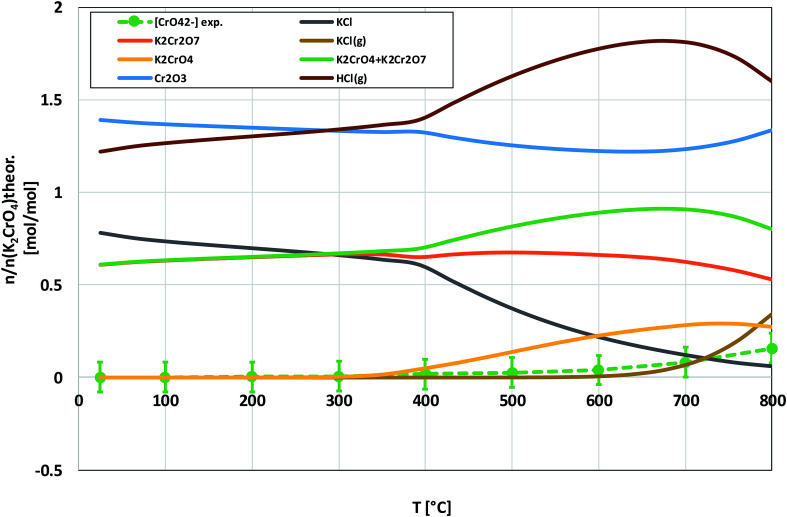
Comparison of the experimentally determined amount of [CrO_4_^2−^] in the filtrates with the thermodynamically predicted amount of the reaction products in the KCl–Cr_2_O_3_ system with pure water leaching.

Compared to KOH, KCl is substantially less reactive with Cr_2_O_3_. As shown in the XRD analysis in Fig. 4, there were no clear signs of reaction with Cr_2_O_3_ until the highest firing temperature, 800 °C. At 800 °C, an additional peak was detected at 26.95° (2*θ*). This peak coincides with the (021) reflection of K_2_Cr_2_O_7_. However, using only one peak in phase identification is not reliable, but based on the analogy with results from the KOH–Cr_2_O_3_ and K_2_CrO_4_–Cr_2_O_3_ systems, K_2_Cr_2_O_7_ was considered to be the most likely reaction product at 800 °C. CrO_4_^2−^ was detected qualitatively and quantitatively also at lower firing temperatures as shown in Fig. 4 and 5. Thermodynamic calculation predicted again the formation of K_2_Cr_2_O_7_ and K_2_CrO_4_ as the major products, but now the discrepancy between the thermodynamic prediction and experimental results was higher than with KOH. CrO_4_^2−^ was detected only in samples exposed to firing temperatures ≥ 400 °C and even at 800 °C the CrO_4_^2−^ amount was only ≈16% of the theoretical maximum.

### 3.4 KOH–Fe_2_O_3_ system

**Fig. 6:**
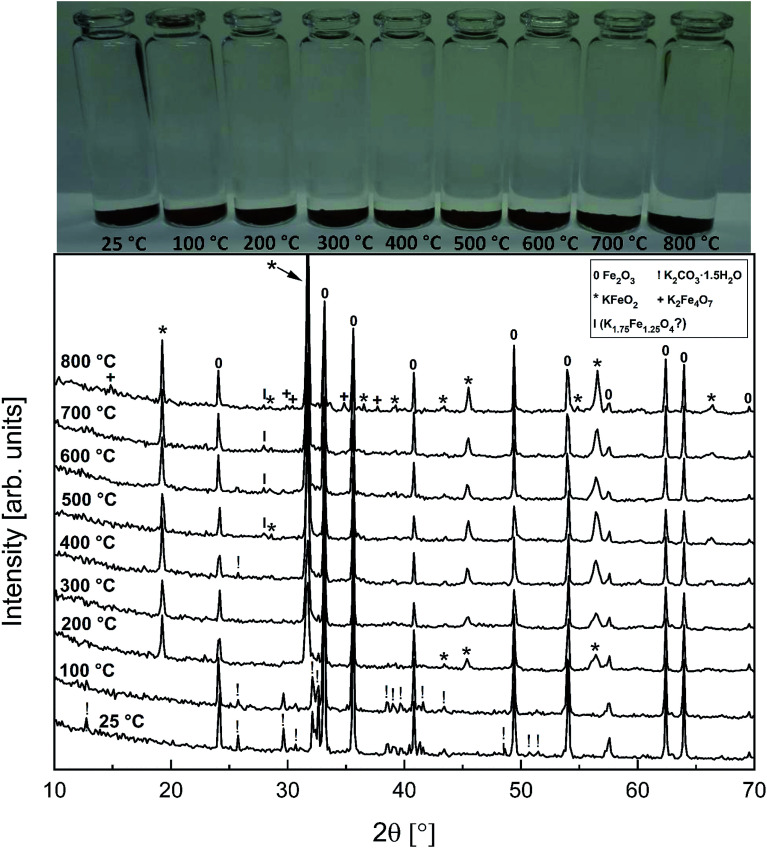
XRD patterns and appearance of the water solutions on top of the KOH–Fe_2_O_3_ mixtures after furnace exposures.

**Fig. 7:**
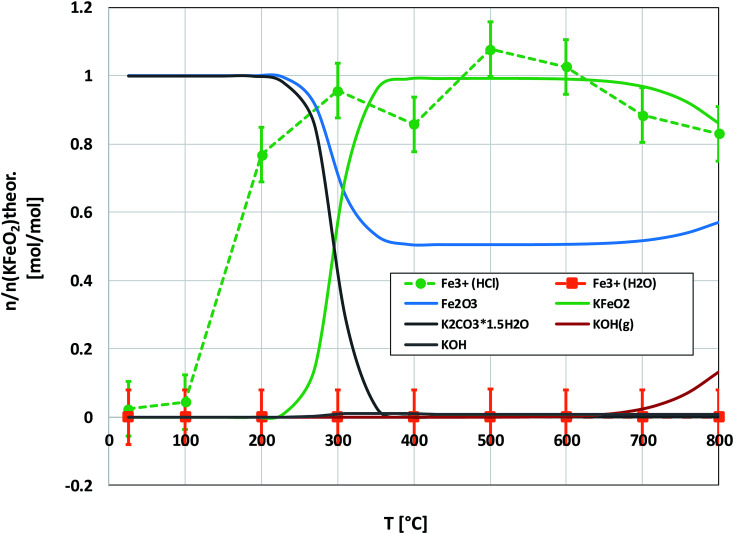
Comparison of the experimentally determined amount of [Fe^3+^] in the filtrates with the thermodynamically predicted amount of the reaction products in the KOH–Fe_2_O_3_ system with pure water leaching and leaching in 1 M HCl_(aq)_ solution.

Fig. 6 shows the XRD analysis of the KOH–Fe_2_O_3_ system. Reaction with Fe_2_O_3_ started at 200 °C firing temperature, forming KFeO_2_ as the reaction product. KFeO_2_ stayed as the major reaction product throughout the temperature range. At 500 °C an additional peak at 27.95° (2*θ*) was detected that could not be assigned to KFeO_2_. This peak was assigned to potassium containing magnetite, K_1.75_Fe_1.25_O_4_ that has the maximum intensity powder XRD peak at this position, but with one peak only, the identification remains doubtful. At 800 °C firing temperature, K_2_Fe_4_O_7_ could also be identified as the reaction product in addition to KFeO_2_. There was no indication of higher oxidation state than +3 reaction products of Fe, such as K_2_FeO_4_. This was consistent with the lack of any colour of the water solutions containing the dissolved reaction products shown in Fig. 6. This result reflects the more favourable tendency of Cr to adapt oxidation state +6 compared to Fe. The comparison of the measured water soluble and acid soluble Fe^3+^ amounts with the thermodynamic prediction are presented in Fig. 7. No water soluble Fe^3+^ was found, but the amount of acid soluble Fe^3+^ was very close to the thermodynamically predicted amount of KFeO_2_. This difference in the filtrates as a function of pH is caused by the fact that either KFeO_2_ is not soluble in water, or that after initial dissolution of KFeO_2_, rapid formation of insoluble Fe(OH)_3_ takes place in the basic solution. The high OH^−^ concentration precipitates the initially dissolved iron and captures it in the filtration residue. In acidic conditions, Fe(OH)_3_ formation is prevented and KFeO_2_ was dissolved completely in the 15 min leaching step and the dissolved iron stayed in the solution phase during the filtration step.

### 3.5 KCl–Fe_2_O_3_ system

**Fig. 8:**
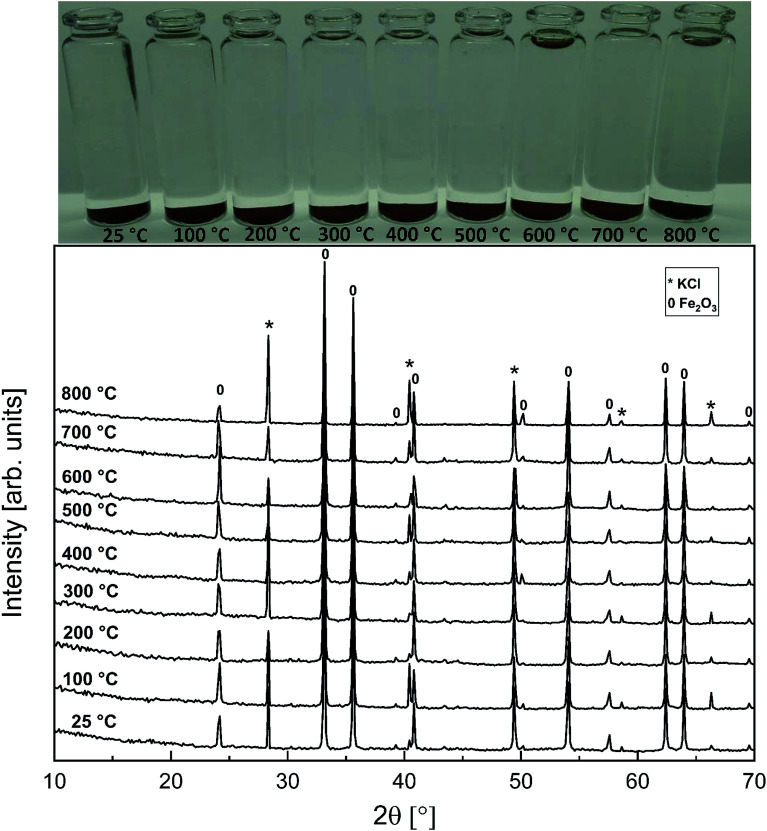
XRD patterns and appearance of the water solutions on top of the KCl–Fe_2_O_3_ mixtures after furnace exposures.

**Fig. 9:**
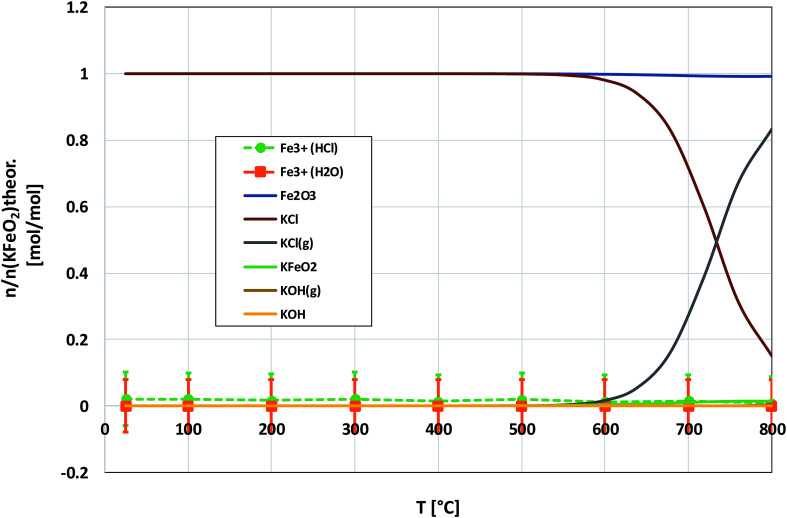
Comparison of the experimentally determined amount of [Fe^3+^] in the filtrates with the thermodynamically predicted amount of the reaction products in the KCl–Fe_2_O_3_ system with pure water leaching and leaching in 1 M HCl_(aq)_ solution.

As with the KCl–Cr_2_O_3_ system, KCl turned out to be much less reactive towards Fe_2_O_3_ compared to KOH. There was no sign of any reaction in the XRD analysis or in the visual qualitative analysis of the water soluble reaction products as shown in Fig. 8. Similar to the KOH–Fe_2_O_3_ case, no water soluble iron was found, but now there was only traces of Fe^3+^ detected even in the acid soluble filtrate. The results agree with thermodynamic equilibrium calculations, which predicted only slight reaction in the KCl–Fe_2_O_3_ system at temperatures > 600 °C to form KFeO_2_ as shown in Fig. 9.

## 4. Discussion

Our results are consistent with the suggestion that the reaction of KCl with Cr_2_O_3_ is initiated by K_2_CrO_4_ formation as found in the corrosion literature.^22,23,43^ However, we argue that the suggested overall reaction of KCl with Cr_2_O_3_:R1
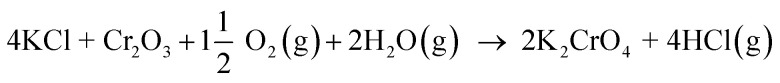
starts with the initiation step:

R2 KCl + H_2_O(g) ↔ KOH + HCl(g)

and then the formed KOH can easily react with the protective oxide as shown in the experiments. Previous hypotheses of the mechanisms of steel corrosion under KCl(s) exposures have focused on the effect of Cl_2_(g) or HCl(g) as the corrosive substances and the effect of KOH has been neglected.^44,45^ However, combustion environments contain inevitably also H_2_O(g) in the flue gases and therefore KOH formation should not be neglected. Furthermore, many virgin biomass fuels contain very little Cl, so there is simply not much Cl_2_(g) or HCl(g) available in the flue gases when combusting these types of fuels. Still KCl layer has been found on the heat exchanger surfaces and previous work has argued that the corroding Cl_2_(g) or HCl(g) must form from the KCl layer, but the fate of the K when the Cl_2_(g) or HCl(g) forms from KCl has not been studied in detail. In waste incineration, the situation is different, because the feedstock contains typically large amounts of Cl and therefore the direct gas phase attack by Cl_2_(g) or HCl(g) may be more relevant. It may be that the chlorine corrosion mechanisms taking place in high chlorine content feedstock combustion cannot be applied to explain the corrosion in virgin biomass combustion.

### 4.1 Reaction of KOH with Cr_2_O_3_

KOH will react readily with Cr_2_O_3_, reacting already at 200 °C:R3
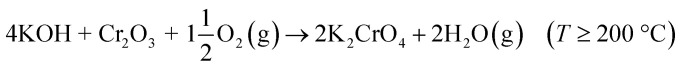


The formed K_2_CrO_4_ can react further to K_2_Cr_2_O_7_ at high temperatures. Direct formation of K_2_Cr_2_O_7_ without the K_2_CrO_4_ intermediate is always kinetically hindered:R4
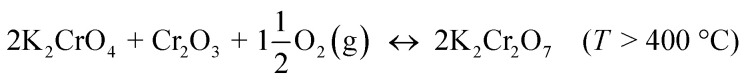


### 4.2 Reaction of KOH with Fe_2_O_3_

KOH will react readily with Fe_2_O_3_, reacting already at 200 °C:

R5 2KOH + Fe_2_O_3_ → 2KFeO_2_ + H_2_O(g) (*T* ≥ 200 °C)

Although not experimentally verified in this study, we propose that in analogy to KOH–Cr_2_O_3_ case, the formed KFeO_2_ can react further to K_2_Fe_4_O_7_ at high temperatures. It is suggested that K_2_Fe_4_O_7_ formation without the KFeO_2_ intermediate is kinetically hindered as the K_2_Cr_2_O_7_ formation in the Cr_2_O_3_ case:

R6 2KFeO_2_ + Fe_2_O_3_ ↔ K_2_Fe_4_O_7_ (*T* > 700 °C)

### 4.3 Reaction of KCl with Cr_2_O_3_ and Fe_2_O_3_

**Fig. 10:**
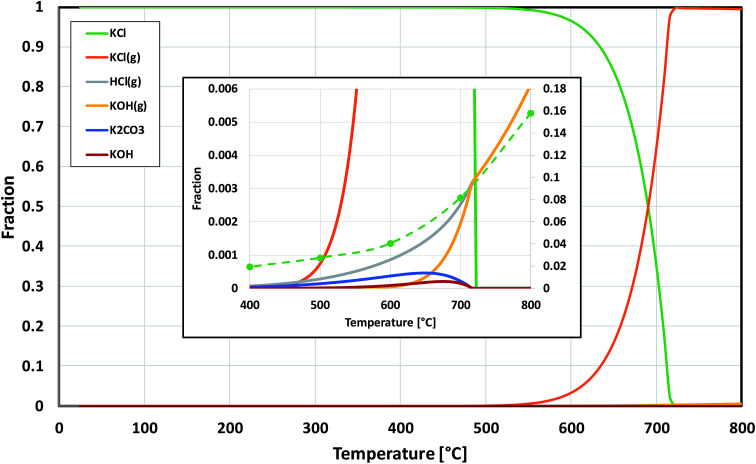
Thermodynamic stability of KCl in humid air, calculated with HSC v6.12 software. The inset is a zoomed image from the bottom right corner of the chart. The green dashed line in the inset shows the trend of the experimentally determined amount of potassium reacted to K_2_CrO_4_ when KCl and Cr_2_O_3_ reacted in the furnace (shown also in Fig. 5).

**Fig. 11:**
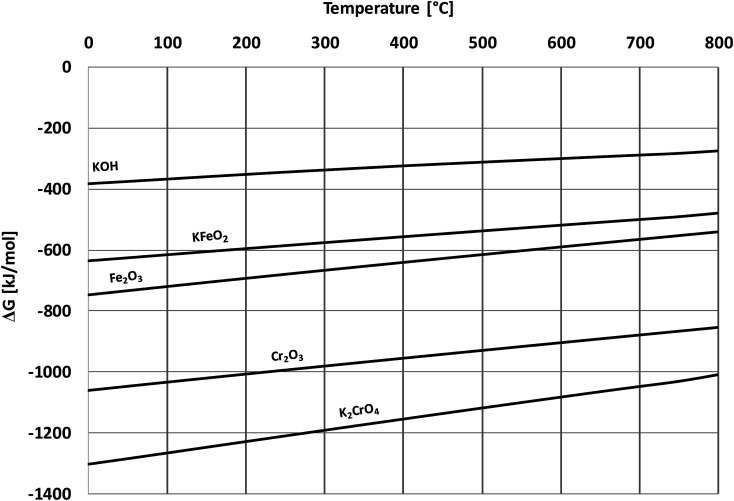
Comparison of Gibbs free energies of formation of the reactants and the corrosion products, calculated with HSC v6.12 software.

Based on the results in this study, it is proposed that the reactivity of KCl towards both oxides in H_2_O(g) containing environment can simply be explained by the thermodynamics of the decomposition reaction R2. Fig. 10 presents the thermodynamic equilibrium of R2 in ambient moist air. It is suggested that HCl(g) does not play a key role in the reaction with the oxides and that the reactive compound is the formed KOH. The reason that we did not detect any reaction between KCl and Fe_2_O_3_, but did detect K_2_CrO_4_ formation with Cr_2_O_3_ at >500 °C can be explained by the higher thermodynamic stability of K_2_CrO_4_ compared to KFeO_2_ as shown in Fig. 11. K_2_CrO_4_ is much more stable than KFeO_2_, therefore it is suggested that the KOH formed in the decomposition of KCl at >500 °C does not form KFeO_2_ as readily as K_2_CrO_4_ and most of the formed KOH is lost in the gas phase by vaporization before reacting with Fe_2_O_3_. That is why KFeO_2_ was not detected in the KCl–Fe_2_O_3_ system.

In case of Fe–Cr steel corrosion, once the protective oxide is destroyed by KOH and if the bare metals are exposed to the atmosphere, HCl(g) may take part in the continuation of corrosion:R7
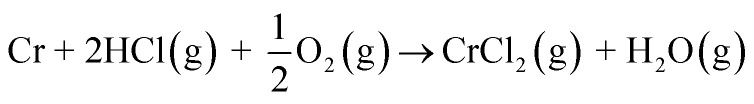
R8
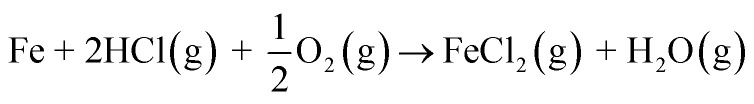
R9
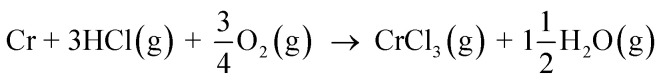
R10
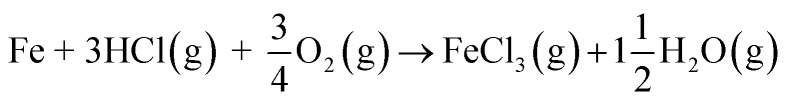


If KCl diffuses through the oxide scale (grain boundary diffusion), the formation of the reactive chlorine species can also take place at the metal-scale interface:

R11 Cr + 2KCl + 2O_2_(g) → K_2_CrO_4_ + Cl_2_(g)

R12 Cr + 2KCl + 4H_2_O(g) → K_2_CrO_4_ + 2HCl(g) + H_2_(g)

R13 2Fe + 2KCl + 2O_2_(g) → 2KFeO_2_ + Cl_2_(g)

R14 2Fe + 2KCl + 2H_2_O(g) → 2KFeO_2_ + 2HCl(g) + H_2_(g)

The formed chlorine compounds can further attack the metal leading to the active oxidation mechanism by chlorine.^46^ However, it is somewhat arbitrary to speculate about the corrosion reactions with elemental metals, because these reactive metals in the elemental form will thermodynamically favour reaction with practically any reactant. For example, a chlorine free corrosion mechanism involving only KOH may also be suggested:R15
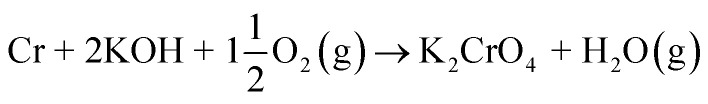
R16
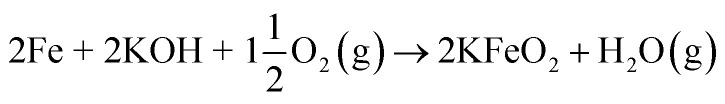


The formed water or the O_2_(g) diffusing through the oxide scale can further oxidize the underlying metal and form Cr_2_O_3_ or Fe_2_O_3_ oxides, which are then consumed by KOH according to reactions R3 and R5, and a chlorine free active oxidation mechanism is established. It is argued that KOH diffuses more readily to the metal scale interphase than KCl, because of the lower melting point of KOH (406 °C) compared to KCl (773 °C) and thus higher mobility at typical service temperatures (400–600 °C). Because of the low melting point of KOH, diffusion can also take place in ionic form by K^+^ and O^2−^ ions diffusing to the metal-scale interphase:

R17 2KOH ↔ K_2_O + H_2_O(g) (in liquid KOH)

R18 K_2_O → 2K^+^ + O^2−^ (in liquid KOH)

R19
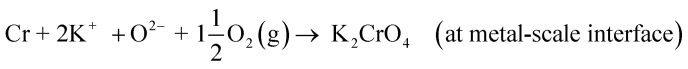
R20
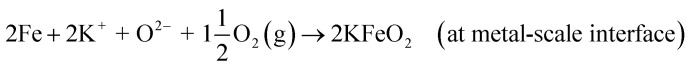


**Fig. 12:**
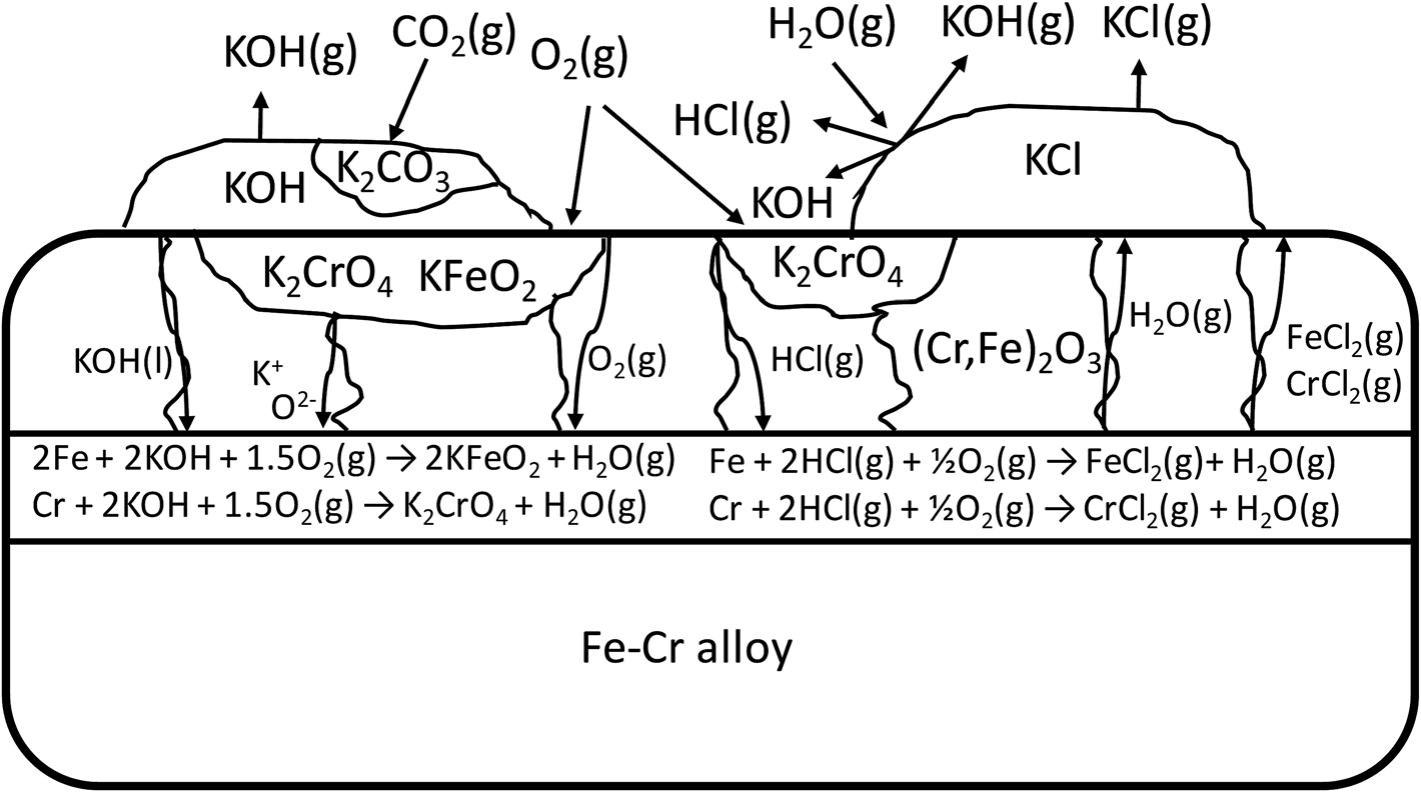
Schematic of the proposed corrosion reactions under KOH and KCl containing salt deposit.

The schematic image in Fig. 12 summarizes the proposed corrosion reactions.

## 5. Conclusions

KOH is much more reactive towards Cr_2_O_3_ and Fe_2_O_3_ than KCl in ambient air environment. Thermodynamic calculations predict the difference in reactivity quite nicely above 400 °C. KOH reacts with both oxides at temperatures higher than 200 °C while KCl reacts with Cr_2_O_3_ at temperatures > 400 °C, but no reaction with Fe_2_O_3_ was detected in the temperature range from room temperature to 800 °C. Both KOH and KCl form K_2_CrO_4_ as the reaction product when reacting with Cr_2_O_3_. K_2_CrO_4_ can further react with Cr_2_O_3_ and form K_2_Cr_2_O_7_ at temperatures > 400 °C. K_2_CrO_4_ and K_2_Cr_2_O_7_ reaction products can be easily leached from the mixture with water at room temperature. KOH forms KFeO_2_ as the reaction product when reacting with Fe_2_O_3_. KFeO_2_ cannot be leached with pure water, but requires an acidic media. In 1 M HCl solution, KFeO_2_ can be easily leached at room temperature. The reactivity of KCl towards the protective oxides of Fe–Cr steels in water containing high temperature environments can be explained by its decomposition to KOH and HCl(g) and the subsequent reaction of the formed KOH with the protective oxide. These results are very valuable for the development of materials for boilers, gasifiers, furnaces and gas turbines utilizing biomass derived fuels as their energy source.

## Conflicts of interest

There are no conflicts of interest to declare.

## Supplementary Material

RA-009-C9RA00582J-s001
