# Supplementary material for: New chemical mechanism explaining the breakdown of protective oxides on high temperature steels in biomass combustion and gasification plants
Source: RSC Adv. 2019 Mar 29;9(18):10034–48. doi: 10.1039/c9ra00582j (PMC9062366; doi:10.1039/c9ra00582j)
Supplement: RA-009-C9RA00582J-s001 [file RA-009-C9RA00582J-s001.pdf]

Electronic Supplementary Material (ESI) for RSC Advances.
This journal is © The Royal Society of Chemistry 2019

High temperature reactions of KCl and KOH with Cr_2O_3 and Fe_2O_3

Tom Blomberg*, Tripurari Tripathi, Maarit Karppinen
Aalto University, Department of Chemistry and Materials Science, Kemistintie 1, 02150 Espoo, Finland
*corresponding author: tom.blomberg@aalto.fi, tripurari.tripathi@aalto.fi, maarit.karppinen@aalto.fi

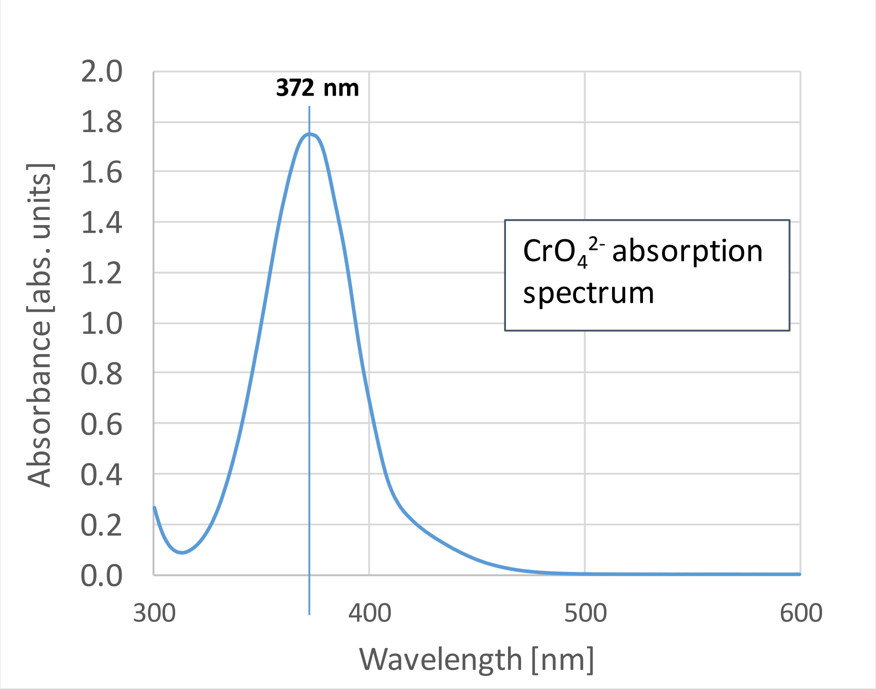

Figure 1. CrO4^{2-} UV/VIS absorption spectrum.

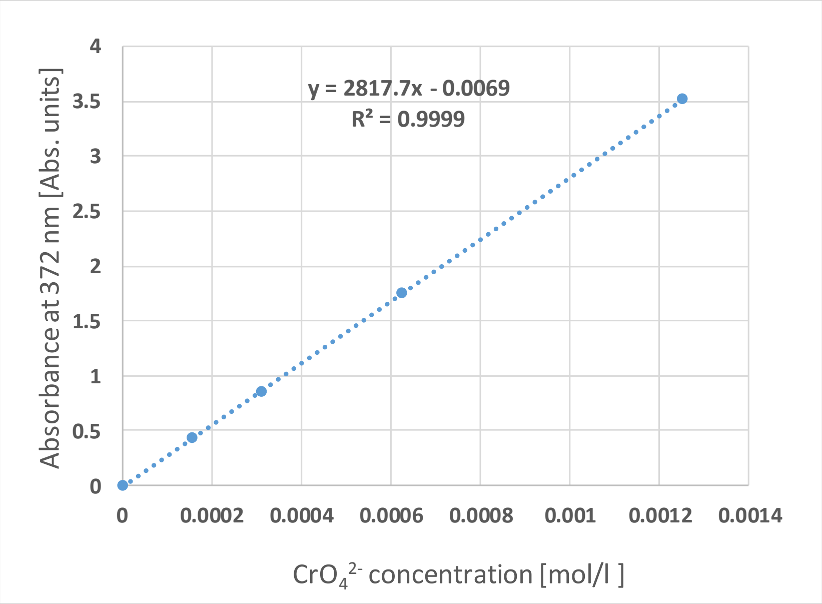

UV_VIS tests_Cr2O3.xlsx

Figure 2. CrO4^{2-} UV/VIS calibration curve.

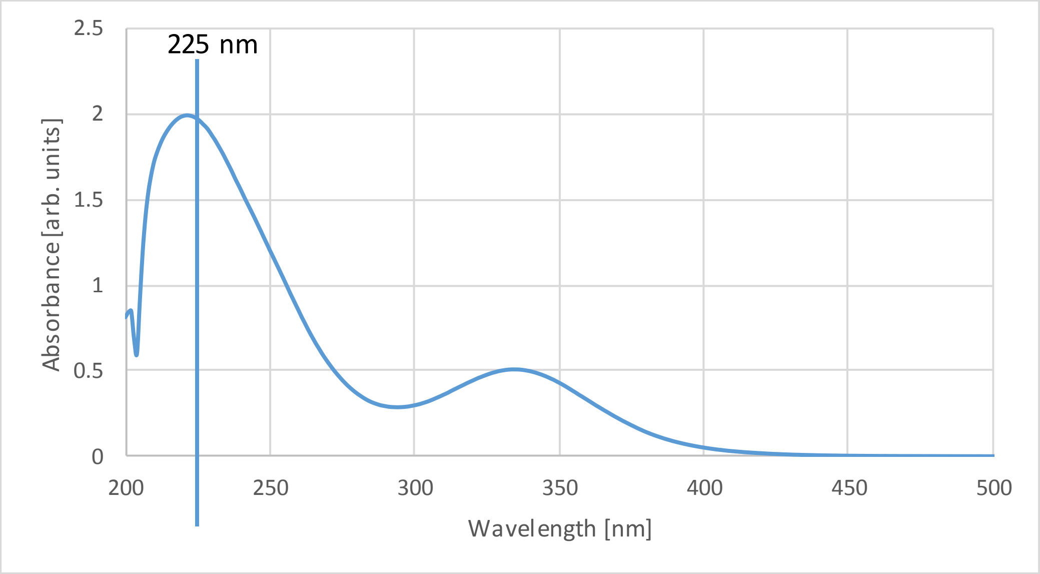

Figure 3. Fe^{3+} UV/VIS absorption spectrum.

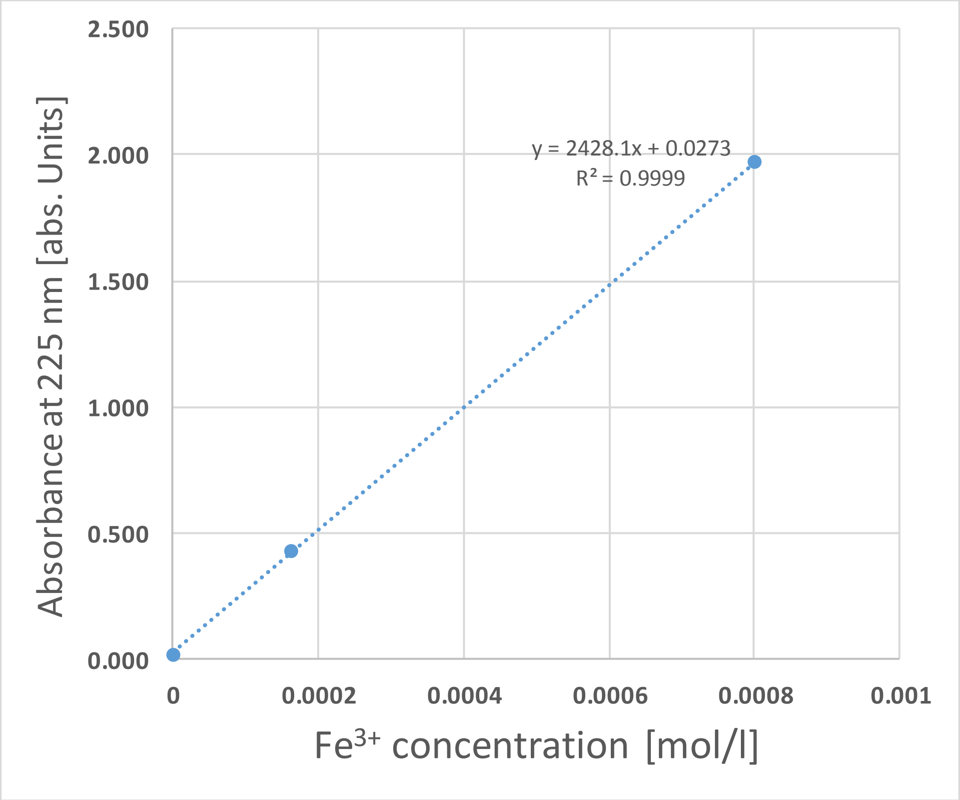

UV_VIS
tests_Fe2O3.xlsx

Figure 4. Fe^{3+} UV/VIS calibration curve.
